# Effects of *Aloe arborescens* Whole Plant Homogenate on Lipid Metabolism, Inflammatory Conditions and Liver Function of Dairy Cows during the Transition Period

**DOI:** 10.3390/ani10050917

**Published:** 2020-05-25

**Authors:** Matteo Mezzetti, Andrea Minuti, Massimo Bionaz, Fiorenzo Piccioli-Cappelli, Erminio Trevisi

**Affiliations:** 1 Department of Animal Sciences, Food and Nutrition (DIANA), Facoltà di Scienze Agrarie, Alimentari e Ambientali, Università Cattolica del Sacro Cuore, 29122 Piacenza, Italy; matteo.mezzetti@unicatt.it (M.M.); andrea.minuti@unicatt.it (A.M.); Fiorenzo.piccioli@unicatt.it (F.P.-C.); 2 Department of Animal and Rangeland Sciences, Oregon State University, Corvallis, OR 97331, USA; massimo.bionaz@oregonstate.edu

**Keywords:** acute phase response, anti-hyperlipidemic, anti-inflammatory, kidney function, nutraceutical

## Abstract

**Simple Summary:**

This study highlights the positive effect of an *Aloe arborescens* Mill. whole plant homogenate on the liver and renal function of dairy cows during the peripartum period. Such positive effects could depend on both anti-hyperlipidemic and anti-inflammatory effects of *Aloe* that could have mitigated hepatic stresses that typically occur in early lactation. Our findings suggest *Aloe arborescens* supplementation to be an effective strategy to ameliorate adverse metabolic conditions in transition cows, indicating it as a preventive nutraceutical strategy against metabolic disorders.

**Abstract:**

The anti-hyperlipidemic and anti-inflammatory effects exerted by *Aloe* on monogastric mammals suggest it as a potential strategy to address the tremendous metabolic alterations that affect dairy cows during their transition to calving. A group of 20 multiparous Italian Holstein dairy cows were housed in freestalls and allocated into two homogeneous groups to receive either 200 g/d of water (CTR) or 200 g/day of *Aloe arborescens* Mill. whole plant homogenate through a rumen tube (AAM) between −14 and 14 days from calving (DFC). From −14 to 35 DFC, the BCS, and milk yield were measured, and blood samples were collected to assess the hematochemical profile. Data underwent ANOVA testing using a mixed model for repeated measurements, including the treatment and time and their interactions as fixed effects. Compared to CTR cows, AAM cows had a less pronounced BCS loss in early lactation (*p* < 0.01), indicating less mobilization of body reserves. Compared to CTR cows, AAM cows had a lower plasma concentration of nonesterified fatty acids and beta hydroxybutyrate (*p* < 0.01 and = 0.01 respectively) that, paired with the lower butterfat content and fat/protein ratio in their milk (*p* = 0.03 and < 0.01 respectively), indicates that *Aloe* reduced the mobilization of body fats. AAM cows had a reduced concentration of myeloperoxidase in plasma and a lower SCC in milk compared to CTR cows (*p* = 0.02 for both), indicating an anti-inflammatory effect of *Aloe*. Furthermore, AAM cows had a lower plasma concentration of ceruloplasmin (*p* < 0.05) and higher plasma concentration of cholesterol, retinol, and paraoxonase compared to CTR cows (*p* < 0.01, < 0.01 and < 0.05 respectively), indicating *Aloe* was effective in mitigating the acute phase response in early lactation. Finally, AAM cows had lower plasma creatinine concentrations around calving (*p* < 0.05), a lower concentration of plasma bilirubin, and a higher concentration of plasma tocopherol compared to CTR cows (*p* = 0.01 for both). These data suggest *Aloe* has anti-hyperlipidemic and anti-inflammatory effects on transition dairy cows that could have ameliorated liver and kidney function disruption and increased the availability of body antioxidants in early lactation.

## 1. Introduction

The transition period (TP) is the most critical phase of a dairy cow’s life [1,2]. Alterations in energy metabolism in this phase lead to a massive mobilization of lipid resources [3] that is often accompanied by systemic inflammation [4,5]. These phenomena result in impaired liver function [6,7] and alter the redox balance in early lactation [8,9].

For millennia, *Aloe* has been used in human medicine in both topical and oral applications [10,11] due to its therapeutic proprieties [12,13] and positive effects on inflammation [14,15,16] and energy metabolism [17]. Previous research attributed such positive effects to many active compounds in both the inner leaf and leaf parenchyma of *Aloe* plants [18,19]. Aloin A is the most studied active compound of *Aloe* and is well known for its antioxidant, antimicrobial, anti-inflammatory, immune-stimulant, and anti-bacterial properties [19,20]. Previous experiments, which fed up to 1000 mg/kg body weight of *Aloe barbadensis* juice extract to murine models for 7 days, suggested the possible use of high dosages without negative effects on liver and kidney functions [21]. Similar experiments performed on diabetic rats [22] found *Aloe* to exert anti-diabetic effects by improving plasma insulin and reducing blood glucose concentration in fasting conditions and to manage dyslipidemia by restoring normal levels of lipoproteins and normal fatty acid composition in the liver and kidney. Other studies confirmed such an anti-hyperlipidemic effect [23] and found *Aloe* to reduce lipid peroxidation, inducing regenerative histological changes in the liver and kidney of diabetic rats [24]. 

Although previous experiments have been mostly performed on monogastric animals, these positive effects on energy metabolism and liver function suggest *Aloe* supplementation as a potential strategy to optimize the transition to calving in dairy cows. In a previous experiment [25], an *Aloe arborescens* Mill. whole plant homogenate (WPH) was ineffective in altering rumen fermentation in vitro, and no effect was detected on feed digestibility and feed intake when supplemented to dairy cows as a drench. However, in the same experiment, Aloin A was detected in the blood of dairy cows as early as 2 h after administration. The lack of any negative effect on feeding behavior, paired with the absorption of bioactive compounds into the blood, opened a new perspective on the use of *Aloe* spp. as a feed supplement in dairy cows, encouraging further investigation of the effects the plant exerts on dairy cow metabolism. We hypothesize that *Aloe* spp. has a positive effect on energy metabolism and inflammation and, as a consequence, improves performance and liver function in early lactation. We assess our hypothesis by supplementing dairy cows between −14 and 14 days from calving (DFC) with a drench of 200 g/d of *Aloe arborescens* Mill. to assess the effects on body condition score (BCS) gain, milk yield (MY), milk composition, and blood profile.

## 2. Materials and Methods

### 2.1. Experimental Design and Animal Management

The trial was carried out at the Università Cattolica del Sacro Cuore research dairy barn (Cerzoo Experiment Station, San Bonico, Piacenza, Italy) in accordance with Italian laws on animal experimentation (DL n. 116, 27 January 1992) and ethics (authorization of the Italian Ministry of Health N 65427; 29 October 2010-PR). From October 2014 to April 2015, a group of 20 multiparous Italian Holstein dairy cows (number of lactations: 3.1 ± 1.2; body weight: 711 ± 53 kg; BCS: 2.51 ± 0.28; MY in the last lactation: 12,005 ± 1384 kg; average lactation length: 350 ± 51 d [mean ± SD]) were enrolled during their dry period and moved to a close-up packed pen 1 month before they were expected to calve. After calving, cows were immediately moved to an early lactation freestall pen until the end of the experiment.

All cows were milked twice a day at 0130 and 1330. Animals were fed a total mixed ration (TMR), formulated in accordance with NRC (2001) [26] protein requirements and with INRA (1989) [27] energy requirements (Table 1).

**Figure 1:**
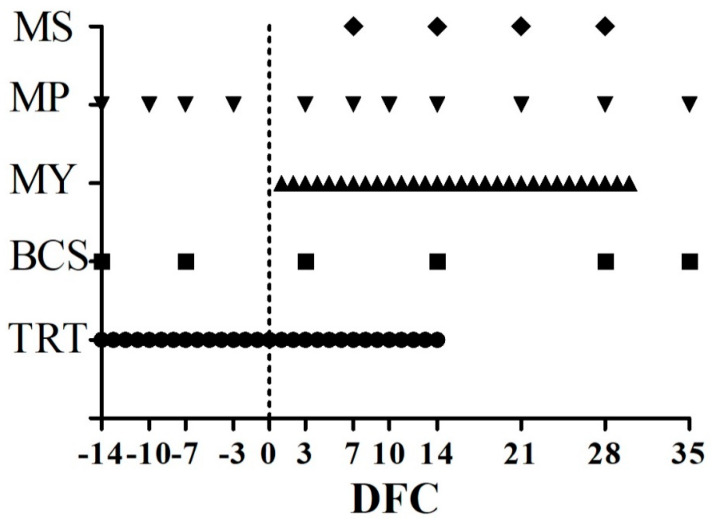
Scheduled time points, expressed as day from calving (DFC), for *Aloe arborescens* Mill. whole plant homogenate or placebo warm water administration (TRT), body condition score determination (BCS), and milk yield measurements (MY), blood sample collection for the metabolic profile (MP) and milk sample collection (MS).

The TMR was distributed once daily at 0830 and 1030 for lactating and dry cows, respectively. A 3% to 5% refusal was guaranteed to ensure cows had ad-libitum access to feed. Representative samples of hay, corn silage, and concentrate were collected monthly. Samples were pooled and analyzed to assess the chemical composition of the feed, as previously described [25]. Analysis results were used to calculate the nutritional values of feed, in accordance with NRC (2001) guidelines [26]. Cows were allocated to one of two homogeneous groups, one receiving 200 g/day of water (CTR) and one receiving 200 g/day of *Aloe arborescens* Mill. WPH between −14 and 14 DFC (AAM). Each dose of WPH was defrosted with warm water (37 °C) before use and water supplemented to the CTR group was heated at the same temperature. Both treatments were administered with a rumen tube before the morning feeding. Periodical checks were performed between −14 and 35 DFC, as shown in Figure 1 and described in the following sections.

### 2.2. Aloe arborescens Mill. Whole Plant Homogenate Preparation

Whole plants of *Aloe arborescens* Mill. (Dester Gardens, Crociale di Manerba del Garda, BS, Italy) were cut and homogenized with a commercial vegetable cutter (model R6, Robot Coupe, Vincennes Cedex, France). Homogenate was immediately frozen in plastic bags, with no additives. Detailed methods for preparation and chemical composition of the *Aloe arborescens* Mill. WPH have been reported previously [25].

### 2.3. Body Condition Score and Milk Yield

The BCS was determined by the same operator using a 1 to 4 scale (Agricultural Development and Advisory Service, 1986) at −14, −7 (SD ± 2 d in all the two time points), 3, 14, 28 and 35 DFC (SD ± 1 d in all the four time points). The MY was measured in the milking parlor by using the Afikim system (SAE Afikim, Kibbutz Afikim, Israele) at each milking between 1 and 30 DFC.

### 2.4. Milk Sample Collection and Analysis

Milk samples were collected from the morning milking at 7, 14, 21, and 28 DFC. The milk composition (fat, protein, lactose, casein content and titratable acidity) was assessed with infrared instrumentation (MilkoScan FT 120, Foss Analytics, Hillerød, Denmark), as previously reported [28]. The fat and protein yields and the fat to protein ratio were also calculated. Urea nitrogen was determined in skimmed milk by a spectrometric assay, using the urea nitrogen kit (cat. No. 0018255440, Instrumentation Laboratory, SpA, Milano, Italy) and the clinical autoanalyzer ILAB-650 (Instrumentation Laboratory, Lexington, Massachusetts, United States). The true protein value was calculated as the difference between the protein and urea nitrogen content. The somatic cell count (SCC) was determined using an optofluorometric method with an automated cell counter (Fossomatic 180, Foss Analytics, Hillerød, Denmark) and expressed as a linear score in accordance with Wiggans and Shook (1987) [29].

### 2.5. Blood Sample Collection and Analysis

Before the morning feeding, blood was collected from the jugular vein at −14 (before treatment administration), −10, −7 (SD ± 2 d in all the three time points), −3 (±1 d), 3, 7, 10, 14, 21, 28 and 35 DFC with 10-mL evacuated heparinized tubes (BD Vacutainer, BD Diagnostics, Franklin Lakes,~NJ,~USA). Samples were processed and analyzed in accordance with Calamari et al. (2016) [30]. 

A clinical autoanalyzer (ILAB-650, Instrumentation Laboratory, Lexington, MA, USA) was used to measure the concentration of glucose, non-esterified fatty acids (NEFA), beta hydroxybutyrate (BHB), urea, creatinine, Ca, Zn, haptoglobin, ceruloplasmin, total protein, albumin, globulin, cholesterol, total bilirubin, aspartate aminotransferase (AST-GOT) and gamma glutamyl transferase (GGT) according to Calamari et al. (2016) [30]. Furthermore, the same instrument was used to determine paraoxonase concentration according to Bionaz et al. (2007) [8], myeloperoxidase according to Bradley et al. (1982) [31], and total reactive oxygen metabolites (ROMt) according to Trevisi et al. (2015) [32]. Retinol, tocopherol, and β-carotene were analyzed by reverse-phase high-performance liquid chromatography (LC-4000, Jasco Europe, Carpi, MO, Italy) as previously described [32]. Further details on the analytical procedures adopted in blood analysis are reported in Table S1.

### 2.6. Statistical Analysis

Data were analyzed in SAS software, version 9.4 (SAS Inst. Inc., Cary, NC, USA) and are presented in graphs and tables as means and pooled standard error for individual means of treatments over time. Before analysis, the normality of distributions was verified for each parameter by reckoning the skewness and kurtosis according to the Shapiro test of SAS. Non-normally distributed parameters were normalized through natural logarithms (among plasma parameters: glucose, BHB, AST-GOT, and bilirubin concentrations; among milk parameters: butterfat, protein, and urea contents and the fat to protein ratio). Original values for log-transformed parameters are presented in Figure S1. Data on BCS, MY and milk and plasma parameters underwent ANOVA testing using a mixed model for repeated measures (Mixed Procedure, SAS Inst. Inc., Cary, NC, USA) in accordance with Littell et al. (1998) [33]. The statistical model included the fixed effect of the treatment (TRT) for CTR and AAM groups, time (considering a single DFC as a repeated measure for each cow) and the interaction of the two factors (TRT × time). The analysis was carried out using two covariance structures: autoregressive order and spatial power, with their heterogeneous counterparts. The compound symmetry has also been tested for parameters collected with equal-spaced time intervals. Covariance structures were ranked according to their Akaike information criterion, with the one having the lowest criterion being chosen [33]. A preliminary analysis was conducted on BCS and blood parameters. These were covariate using data collected at −14 DFC as the baseline, adopting *p* ≤ 0.1 as a cutoff for covariate inclusion. None of the parameters had a significant covariate effect in the preliminary analysis and the covariate was thus removed from the final model. The pairwise comparison was done using the least significant difference test. Post-hoc comparisons were discussed when the *p*-value for the main effect was 0.05 or less. The main effects at *p* ≤ 0.1 were discussed in the context of tendencies.

## 3. Results

### 3.1. Body Condition Score and Milk Yield

**Figure 2:**
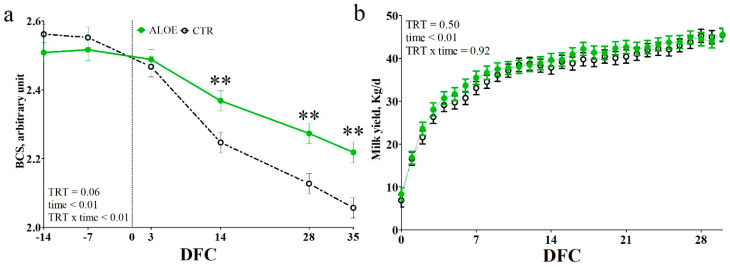
Values of BCS (**a**) and milk yield (**b**) in dairy cows receiving placebo water (CTR; black dotted line) or cows receiving a drench of 200 g/d of *Aloe arborescens* Mill. whole plant homogenate between −14 and 14 days from calving (AAM; green solid line). ** is *p* < 0.01; DFC is days from calving.

The BCS tended to be higher in AAM cows compared to CTR cows (*p* = 0.06; Figure 2a), and a TRT × time interaction was also observed (*p* < 0.01). Both groups had a similar BCS before calving and till 3 DFC, but the AAM cows had higher BCS than CTR cows from 14 DFC to the end of the experimental period (*p* < 0.01 at each time point). No difference between groups was detected for the MY (Figure 2b).

### 3.2. Milk Quality Parameters

Regarding milk quality parameters (Table 2), AAM cows had a lower butterfat content, fat/protein ratio and SCC compared to the CTR cows (*p* = 0.03, *p* < 0.01 and *p* = 0.02, respectively) but higher casein content than CTR cows (*p* = 0.02). A TRT × time interaction was observed for the butterfat, fat output and fat/protein ratio (*p* = 0.01, *p* = 0.03 and *p* < 0.01, respectively), with AAM cows having the lowest values at 7 DFC (*p* < 0.01). No effect was detected for the other parameters.

### 3.3. Metabolic Profile

#### 3.3.1. Energy and Protein Metabolism Biomarkers, Kidney Function Indicators and Mineral Metabolism Biomarkers

**Figure 3:**
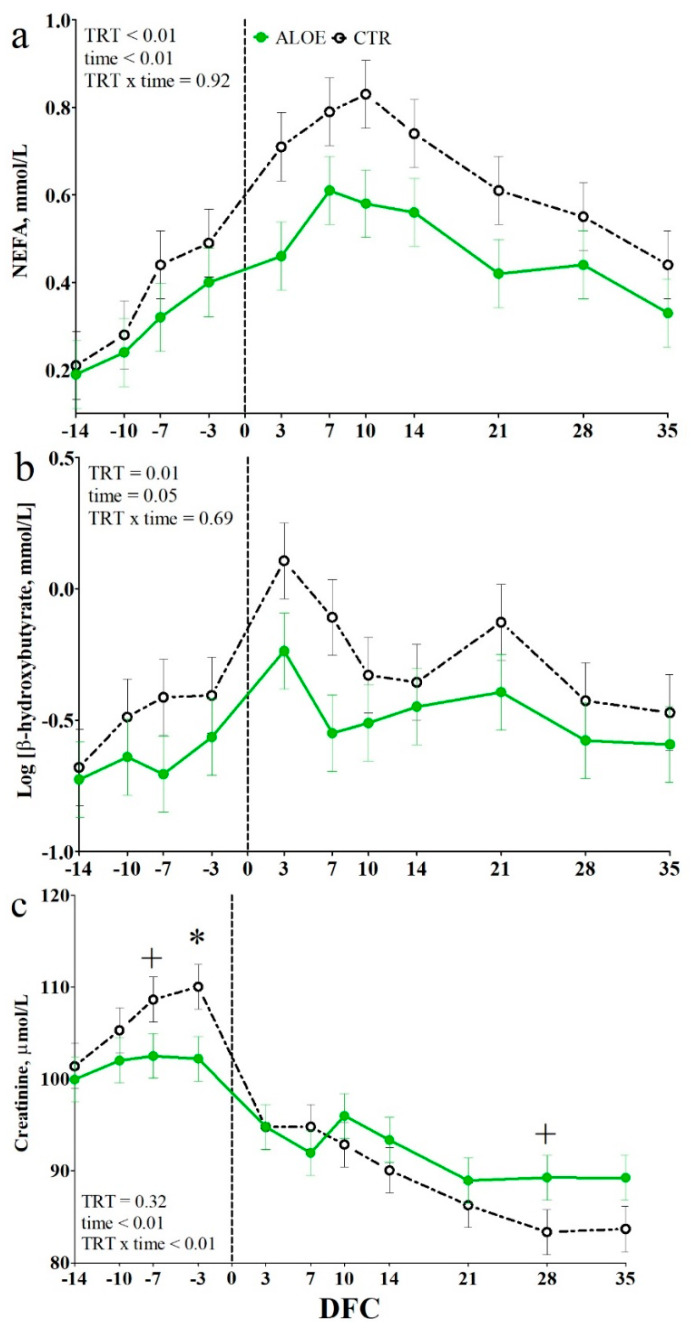
Time course of plasma concentrations of non-esterified fatty acids. NEFA (**a**), beta-hydroxybutyrate (**b**), and creatinine (**c**) in dairy cows receiving placebo water (CTR; black dotted line) or cows receiving a drench of 200 g/d of *Aloe arborescens* Mill. whole plant homogenate between −14 and 14 days from calving (AAM; green solid line). * is *p* < 0.05; + is *p* < 0.1; Log indicates data expressed as log-transformed; DFC is days from calving.

Among the energy metabolism biomarkers, glucose concentration was not affected by TRT (Figure S3a). The concentrations of NEFA and BHB were lower in the AAM cows than in the CTR cows (*p* ≤ 0.01; Figure 3a,b). Among the protein metabolism biomarkers, the urea concentration was not affected by TRT (Figure S3b). Among kidney function biomarkers, creatinine concentration had a significant TRT × time interaction (*p* < 0.01; Figure 3c). In comparison to CTR cows, the AAM cows had a lower creatinine concentration at −7 and −3 DFC (*p* < 0.1 and *p* < 0.05, respectively) and a tendency toward higher creatinine concentration at 28 DFC (*p* < 0.1). Mineral concentration did not differ between the two groups (Figure S3c,d).

#### 3.3.2. Liver Function and Inflammation Biomarkers

**Figure 4:**
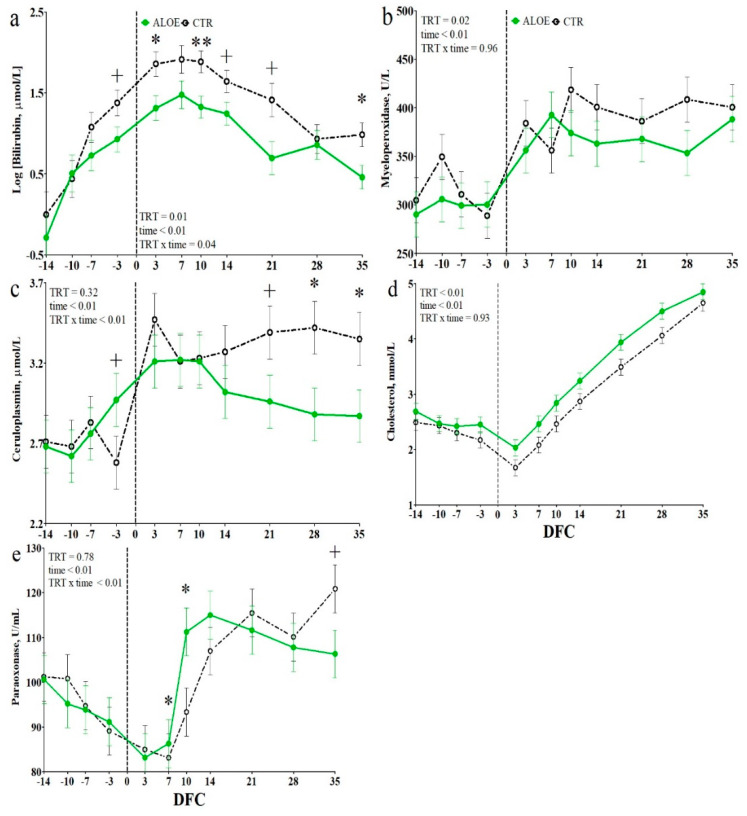
Time course of plasma concentrations of bilirubin (**a**), myeloperoxidase (**b**), ceruloplasmin (**c**), cholesterol (**d**) and paraoxonase (**e**) in dairy cows receiving placebo water (CTR; black dotted line) or cows receiving a drench of 200 g/d of *Aloe arborescens* Mill. whole plant homogenate between −14 and 14 days from calving (AAM; green solid line). ** is *p* < 0.01; * is *p* < 0.05; + is *p* < 0.1; Log indicates data expressed as log-transformed; DFC is days from calving.

Among liver function biomarkers, total bilirubin concentration was affected by TRT and the TRT × time interaction (*p* < 0.01 and *p* = 0.04 respectively; Figure 4a); it was lower in the AAM cows than in the CTR cows between −3 DFC and the end of the experimental period. The GGT concentration had a tendency toward a TRT × time effect (*p* = 0.06; Figure S3e) and was numerically higher in the AAM cows than in the CTR cows before calving. AST-GOT was not affected by TRT (Figure S3f). 

**Figure 5:**
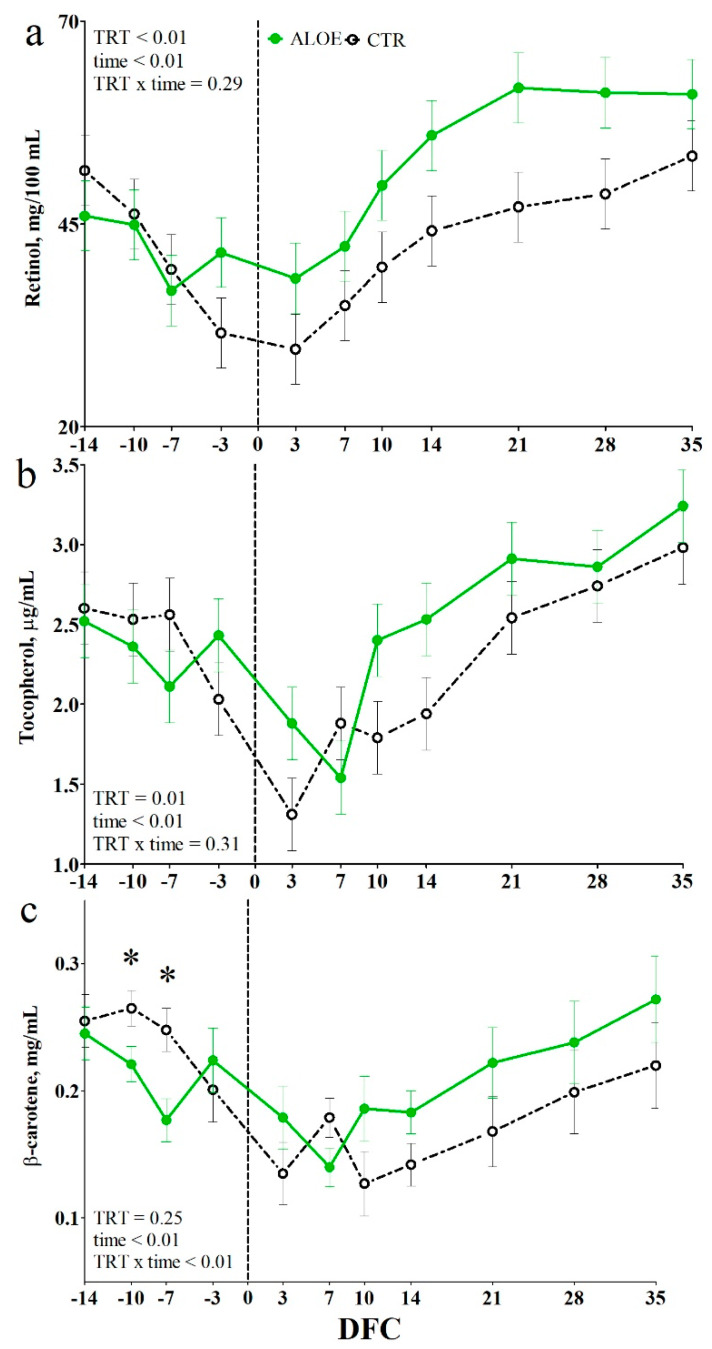
Time course of plasma concentrations of retinol (**a**), tocopherol (**b**), and β-carotene (**c**) in dairy cows receiving placebo water (CTR; black dotted line) or cows receiving a drench of 200 g/d of *Aloe arborescens* Mill. whole plant homogenate between −14 and 14 days from calving (AAM; green solid line). * is *p* < 0.05; DFC is days from calving.

Among the inflammation biomarkers, the concentration of myeloperoxidase was lower in the AAM cows compared with the CTR cows (*p* = 0.02; Figure 4b). No effect was detected for total protein and globulin (Figure S3g,h). Among the positive acute-phase proteins (APP), no effect was detected for the haptoglobin level (Figure S3i), whereas ceruloplasmin had a TRT × time interaction (*p* < 0.01; Figure 4c). Compared to the CTR cows, AAM cows had lower ceruloplasmin concentration from 21 DFC to the end of the experimental period (*p* < 0.05 at each time point). Among the negative APPs, levels of cholesterol and retinol were higher in the AAM cows than in the CTR cows (*p* < 0.01; Figure 4d or Figure 5a), whereas paraoxonase concentration had a TRT × time interaction (*p* < 0.01; Figure 4e) resulting in higher levels in the AAM cows than in the CTR cows at 7 and 10 DFC (*p* < 0.05). No difference between groups was detected for albumin (Figure S3j).

#### 3.3.3. Redox Balance Biomarkers

The concentration of tocopherol was higher in the AAM cows than in the CTR cows (*p* = 0.01, Figure 5b), whereas β-carotene level was affected by a TRT × time interaction (*p* < 0.01, Figure 5c) and was lower in the AAM cows than in the CTR cows at −10 and −7 DFC (*p* < 0.05). Concentrations of ROMt were not different between groups (Figure S3k).

## 4. Discussion

### 4.1. Aloe Reduced Mobilization of Body Reserves and Improved Lipid Metabolism in Transition Period

A negative energy balance is known to affect transition dairy cows, leading to the sudden mobilization of body reserves [3]. The less-marked BCS loss in our AAM cows compared to CTR cows suggests WPH mitigated mobilization processes in early lactation. This is supported by the lower NEFA level found in the plasma of our AAM cows, reflecting a less-marked mobilization of lipid resources [34]. The reduced mobilization of body fats found in our study is consistent with the reduction in triglycerides and free fatty acids found in the plasma, liver, and kidney of murine models of Streptozotocin-induced diabetes and Letrozole-induced polycystic ovarian syndrome after feeding with *Aloe barbadensis* ethanolic extract [22,23]. Although obtained in murine models and using *Aloe barbadensis* instead of *Aloe arborescens*, these anti-hyperlipidemic effects observed against diabetes and polycystic ovary syndrome–related dyslipidemia suggests that the reduced NEFA concentration in the blood of our early lactating cows could depend on the effectiveness of WPH in managing body fat mobilization. 

Phytosterols contained in *Aloe* gel, such as lonophenol and cycloartenol, are peroxisome proliferator-activated receptor alpha (PPARα) and peroxisome proliferator-activated receptor gamma (PPARγ) agonists in monogastric animals [35], and also emodin is known to activate PPARγ [36]. Although the content of these compounds in our WPH has not been assessed and their effective absorption in blood has never been investigated, most of the effects of *Aloe* in our transition cows are consistent with the activation of PPAR signaling; these effects are supported by previous results in murine models [17,23,24]. PPARγ plays a critical role in controlling lipolysis, although the driving mechanism of such interaction is still not fully elucidated. On one hand, the key lipolytic enzyme adipose triglyceride lipase (*ATGL*) is a PPARγ target gene [37]; however, lipolysis is inhibited during adipogenesis [38], which is mostly driven by activation of PPARγ [39]. Thus, there are two possible mechanisms by which PPARγ inhibits lipolysis in adipose tissue: It increases insulin sensitivity [40], which potently inhibits lipolysis [38], and it decreases the activity of *ATGL* via perilipin, a PPARγ target [41]. The reduction of NEFA observed in our study can also be driven by the activation of PPARα. This transcription factor is known to control the expression of genes involved in the entry of NEFA into mitochondria [42]. Thus, we can speculate that the lower NEFA level in our AAM cows could partially depend on faster removal from the bloodstream paired with an increased insulin sensitivity affected by several secondary compounds present in *Aloe*, as recently reviewed [43]. 

In dairy cows, a NEFA overload in the liver could impair the β-oxidation process [44,45], leading to the release of ketone bodies (as BHB) in the blood [34].The lower BHB level in the blood of our AAM cows could thus result from their lower amount of circulating NEFA, but it could also partially depend on the effectiveness of *Aloe* in restoring the normal activity of lipid-metabolizing enzymes [23], which could have improved the oxidative power of the liver in oxidizing NEFA. This effect is also consistent with the activation of PPARα driven by *Aloe* components, which is known to increase fatty acid catabolism by improving β-oxidation [46,47].

Such positive effects of WPH on lipid metabolism are consistent with the lower butterfat, fat output, and fat to protein ratio in the milk of our AAM cows compared to CTR cows; both NEFA and BHB are known to directly contribute to the de novo synthesis of milk fat [48,49]. Despite trends of BCS, NEFA, BHB, and milk fat detected in our AAM cows suggest a direct effect of *Aloe* compounds on lipid metabolism, the lack of individual feed intake and plasma insulin concentration measurements must be pointed out as a limitation of our experimental design. Thus, further investigations including individual feed intake measurement, energy balance calculation, plasma insulin concentration assessment, glucose tolerance test in peripheral tissues, and liver biopsies are required to confirm that lower lipid mobilization found in our cows receiving *Aloe* is driven by a direct effect in ameliorating lipid metabolism through improving insulin sensitivity and NEFA removal from the bloodstream rather than a positive effect on mitigating the negative energy balance through improving feed intake.

### 4.2. Aloe Improved Kidney Function in Late Gestation and Mitigated the Acute Phase Response and Improved Liver Function in Early Lactation

Cows in early lactation are physiologically affected by an acute phase response in the liver [6,50]. During the acute phase, the liver shifts its anabolic priorities; the plasmatic trends of the APPs reflect the severity of the phenomenon [51]. Ceruloplasmin is known as a positive APP, and typically its plasma concentration increases when an acute phase response occurs [52]. Conversely, paraoxonase, lipoproteins, and retinol-binding protein are labeled negative APP because their plasma concentrations typically decrease during an acute phase response [4,8]. The faster decrease in ceruloplasmin and the higher concentration of paraoxonase, cholesterol (as an index of lipoprotein) and retinol (reflecting the plasma trends of retinol-binding protein) observed in AAM cows in early lactation suggest a less inflammatory-like condition or a faster resolution of the acute phase response compared to CTR cows [53]. The less-marked acute phase response in AAM cows fits with the activation of PPARs driven by *Aloe*, as PPARs are potent negative regulators of the acute phase response in different species, including cattle [47]. Such a negative regulation could be a result of an anti-inflammatory effect exerted by *Aloe* through interacting with PPARγ, as suggested by the lower SCC paired with the lower plasma myeloperoxidase concentration.

Immune dysfunction in dairy cows during the TP could lead to uncontrolled inflammation, and such inflammatory conditions are known to worsen the acute phase response in the liver once lactation starts [50,54,55]. The SCC is a widely accepted indicator for local inflammations because it reflects the migration of leukocytes to cope with microbial invasions in the udder [56]. PPARγ is known to downregulate the production of pro-inflammatory cytokines involved in the recruitment of leukocytes to the mammary gland [57], partially accounting for the beneficial effect exerted by *Aloe* in reducing SCC. This is supported by the downregulated expression of the chemoattractant of neutrophils interleukin 8 in mammary macrophages observed in LPS-stimulated human macrophages incubated with *Aloe* [58]. The shift from a pro- to an anti-inflammatory phenotype induced in white blood cells by PPARγ activation could also account for the lower myeloperoxidase concentration found in the blood of our AAM cows compared to CTR cows. Myeloperoxidase is involved in the generation of reactive oxygen species in neutrophils [59], and its plasma concentration could thus serve as a reliable marker of inflammation in the bloodstream. Thus, the reduction of local inflammation could have reduced the likelihood of development of systemic inflammation, as reflected by the lower myeloperoxidase levels found in the blood.

An impairment of renal glomerular filtration rate has been reported in late gestation in many different species, including ruminants [60], and the massive utilization of amino acids for gluconeogenesis could concur in renal dysfunction of dairy cows during the late gestation [61]. Recent studies performed on monogastric models and humans found high levels of circulating pro-inflammatory cytokines (i.e., tumor necrosis factor alpha, interleukin 1) released during excessive lipid mobilization and uncontrolled inflammation to induce renal artery endothelial dysfunction [62,63,64], thus suggesting also high circulating NEFA and systemic inflammations occurring in dairy cows during TP to play a pivotal role in inducing renal dysfunctions in late gestation. Creatinine is related to phosphocreatine utilization during normal muscular activity, and the hematic concentration of this metabolite serves as a reliable index of glomerular filtration efficiency, as it reflects the capacity of the kidneys to remove it [65]. Thus, lower levels of plasma creatinine found in AAM cows before calving suggests WPH has improved their renal glomerular filtration rate as compared to CTR cows, deposing for a positive effect of *Aloe* on kidney function, probably induced by its anti-hyperlipidemic and anti-inflammatory actions. Similarly, the liver function of cows in early lactation is commonly impaired by an overload of NEFA and by the acute phase response [45,53]. Positive effects exerted by *Aloe* on lipid metabolism and inflammatory conditions could thus account for the reduced bilirubin levels found post-partum in the blood of AAM cows compared to CTR cows, indicating improved liver function. Bilirubin is the result of the degradation of red blood cells, and increased concentrations of bilirubin in the blood indicate reduced clearance activity by liver enzymes [66]. 

Such positive effects of *Aloe* in restoring liver and kidney function have been previously reported by Adesokan et al. (2010) and Ramachandraiahgari et al. (2012) [21,24], who observed regenerative liver and kidney histological changes in rats with Streptozotocin-induced diabetes receiving *Aloe barbadensis* or its polysaccharides. More recently, Saka et al. (2011), Nahar et al. (2013), and Koo et al. (2019) [67,68,69] observed a reduction of bilirubin in rats and humans receiving *Aloe* treatments. The reduction of bilirubin before parturition in AAM cows appears to indicate more direct activity of *Aloe* on bilirubin clearance in the liver. The transcription of the main enzyme involved in the conjugation of bilirubin, uridine 5′-diphospho-glucuronosyltransferase, is under the control of several nuclear receptors, including PPAR. Prior studies have determined that phytosterols present in *Aloe vera* are potent PPARα and PPARγ agonists, as observed in grivet kidney cells and confirmed in mouse liver [35]. Thus, the reduction in bilirubin observed in our study is likely due to a transcriptomic effect of *Aloe vera* phytosterols in the liver via PPAR activation. 

Higher tocopherol concentrations found in the blood of AAM cows is not surprising, considering the high level of this vitamin in *Aloe* leaf [70], although positive effects exerted by WPH in mitigating the acute phase response and improving liver function could have contributed to higher tocopherol levels. Tocopherol is known to serve as a secondary antioxidant, reducing the chain propagation and amplification of lipid peroxidation [71], and a massive depletion of body stocks of this vitamin (as well as other antioxidant systems) is known to occur in early lactating cows [72,73]. The liver plays a pivotal role in reducing antioxidant compounds when they have scavenged free radicals and other oxidant species in the body [72,73], and lipoproteins are the main carrier of tocopherol in blood [74]. Thus, both a positive effect of *Aloe* on the activity of liver antioxidant enzymes [24], which could have improved the recovery of oxidized tocopherol, and a greater availability of lipoproteins (as suggested by the higher cholesterol level found in AAM cows), which could have improved the circulation of this vitamin in the blood, could have contributed to higher concentrations of circulating tocopherol in AAM cows compared to CTR cows. Such increased tocopherol availability could have benefited udder health, also accounting for the lower SCC in the milk of cows receiving *Aloe* found in our study. Tocopherol is known to directly regulate tissue integrity, improving the tightness of the Furstenberg’s rosette at the teat end, and thus being beneficial in mastitis control [75].

## 5. Conclusions

In our study, the reduced level of NEFA and BHB in the blood of cows receiving *Aloe* is consistent with their reduced milk fat and could account for their reduced mobilization of body fats and reduced ketogenesis; these effects could be driven by greater efficiency in the oxidation of NEFA at the liver level. The reduced mobilization of body fat in AAM vs. CTR cows, together with the less-marked acute phase response in early lactation, could partly account for the reduced bilirubin level, reflecting improved liver function. Although high concentrations of vitamin E in *Aloe* leaf could largely account for the greater availability of blood tocopherol found in AAM cows in early lactation, ameliorated disruption of liver function could have improved management of antioxidant systems by liver enzymes in this phase, partially accounting for such an effect.

Overall, our data suggest a role for PPARs activation in the metabolic effects exerted by *Aloe* on transition dairy cows. Effects on lipid metabolism are consistent with the activation of PPARγ on adipose tissue and activation of PPARα at the liver level. The positive effects on acute phase indicators could depend on an anti-inflammatory effect of *Aloe* driven by the activation of both PPAR isotypes. Although the interaction between minor *Aloe* compounds and PPARs has already been demonstrated, an in-depth study is required to confirm the absorption of these secondary compounds into the blood. Furthermore, a study taking a transcriptome approach to investigate the alterations induced by *Aloe* on pathways related to energy metabolism and inflammation could highlight the effect of the different compounds in *Aloe* on PPARs activation.

## Figures and Tables

**Table 1:** Composition and characteristics on a daily basis of the experimental diets fed to cow during dry and lactation periods.

	Dry Period	Lactation Period
Item, %DM		
Corn silage	25.0	33.5
Corn grain	-	17.2
Alfalfa hay	-	15.3
Grass hay	51.6	3.5
Soybean meal	7.3	9.2
Corn flakes	-	7.4
Sunflower meal	-	2.4
Corn gluten meal	-	1.7
Supplement ^1^	1.0	2.1
Cotton seeds	-	7.7
Wheat straw	15.3	-
Chemical composition		
NEL, Mcal/kg of DM	1.36	1.54
Crude protein, % DM	12.50	16.00
Starch + sugar, %DM	12.50	29.80
Ether extract, %DM	2.70	4.50
NDF, %DM	54.9	33.70

^1^ 42.9% Ca_2_PO_4_; 28.6% urea; 14.3% MgO; 7.1% NaCl; 7.1% mineral vitamin supplement. The supplement for dry cows was composited to provide 1,500,000 UI/kg of vitamin A, 150,000 IU/kg of vitamin D3, 7000 UI/kg of vitamin E, 1100 mg/kg of Mn, 4000 mg/kg of Zn, 500 mg/kg of Cu, 70 mg/kg of I, 10 mg/kg of Co and 23 mg/kg of Se. The supplement for lactating cows was composited to provide 150,000 UI of vitamin A, 10,000 IU of vitamin D3, 200 mg of vitamin E, 100 mg of vitamin K, 100 mg of vitamin H1, 50 mg of vitamin B1, 0.5 mg of vitamin B12, 500 mg of vitamin PP, 4000 mg of choline, 350 mg of Mn, 800 mg of Zn, 40 mg of Cu, 20 mg of I, 1 mg of Co and 1 mg Se.

**Table 2:** Milk composition and somatic cell count in control dairy cows or cows receiving 200 g/d of *Aloe arborescens* Mill. homogenate extract as a drench between −14 and 14 days from calving.

Item, Unit	TRT ^1^	Days From Calving				SEM ^2^	*p*-Value		
		7	14	21	28		TRT ^1^	Time	TRT × Time ^3^
Butterfat	CTR	5.09	4.24	3.81	3.87	0.34	0.03	0.01	0.01
mg/100 mL	AAM	3.80	4.00	3.77	3.80				
		**							
Fat output	CTR	166.0	159.4	154.4	175.1	11.7	0.33	0.02	0.03
g	AAM	133.7	157.9	160.2	168.1				
		**							
Total protein	CTR	3.91	3.44	3.27	3.20	0.10	0.22	<0.01	0.94
mg/100 mL	AAM	3.99	3.55	3.38	3.26				
								
Protein output	CTR	129.2	130.7	132.0	144.7	21.17	0.25	0.14	0.22
g	AAM	141.1	139.9	143.8	144.4				
								
Fat/protein ratio	CTR	1.32	1.24	1.17	1.21	0.06	<0.01	0.19	<0.01
-	AAM	0.95	1.13	1.12	1.17				
		**							
Lactose	CTR	4.69	4.72	5.00	4.99	0.20	0.61	0.01	0.34
mg/100 mL	AAM	4.81	4.99	5.07	4.76				
								
Caseins	CTR	2.74	2.52	2.40	2.37	0.12	0.02	<0.01	0.76
mg/100 mL	AAM	2.96	2.65	2.53	2.45				
								
Titratable acidity	CTR	4.23	3.53	3.34	3.24	0.24	0.68	<0.01	0.49
°SH/50 mL	AAM	4.09	3.64	3.52	3.44				
								
Urea-N	CTR	22.0	23.0	20.5	21.1	2.60	0.88	0.45	0.47
mg/dL	AAM	22.5	20.5	20.4	21.9				
								
True protein ^4^	CTR	3.69	3.21	3.06	2.99	0.10	0.22	<0.01	0.87
mg/100 mL	AAM	3.77	3.34	3.18	3.04				
								
Somatic cells count	CTR	3.38	3.47	3.27	3.99	1.15	0.02	0.75	0.78
Linear score	AAM	2.33	1.66	1.09	1.36				
								

^1^ Treatment (CTR is cows receiving placebo water; AAM is cows receiving a drench of *Aloe arborescens* Mill.). ^2^ Standard error = larger standard error for the fixed effects. ^3^ Treatment × time interaction (** is *p* < 0.01 for differences among means within a column. These symbols are only presented when the interaction effect is significant). ^4^ True protein = total protein-(urea-N/100).

## Supplementary Materials

The following are available online at https://www.mdpi.com/2076-2615/10/5/917/s1, Table S1: Intra- and inter-assay coefficient of variations, limit of quantification (LOQ), codes of commercial kits used and references for their validation in the bovine plasma for plasma parameters included in the study. Figure S1: Pattern of plasmatic concentrations of glucose (a), beta hydroxybutyrate (b), glutamate-oxaloacetate transaminase (AST-GOT; (c) and bilirubin (d) expressed as their original values in dairy cows receiving placebo water (CTR; black dotted line) or cows receiving a drench of 200 g/d of *Aloe arborescens Mill.* whole plant homogenate between −14 and 14 days from calving (AAM; green solid line). DFC is days from calving. Figure S2: Pattern of plasmatic concentrations of glucose (a), beta hydroxybutyrate (b), glutamate-oxaloacetate transaminase (AST-GOT; (c) and bilirubin (d) expressed as their original values in dairy cows receiving placebo water (CTR; black dotted line) or cows receiving a drench of 200 g/d of *Aloe arborescens Mill.* whole plant homogenate between −14 and 14 days from calving (AAM; green solid line). DFC is days from calving.
