# Supplementary material for: Effects of Aloe arborescens Whole Plant Homogenate on Lipid Metabolism, Inflammatory Conditions and Liver Function of Dairy Cows during the Transition Period
Source: Animals (Basel). 2020 May 25;10(5):917. doi: 10.3390/ani10050917 (PMC7278487; doi:10.3390/ani10050917)
Supplement: Supplementary file 1 [file animals-10-00917-s001.pdf]

Supplementary Materials:

Supplementary file 1. Intra- and inter-assay coefficient of variations, limit of quantification (LOQ), codes of commercial kits used and references for their validation in the bovine plasma for plasma parameters included in the study.

Parameter	Inter	Intra	LOQ	Commercial kit	Reference
Glucose	0.75	1.33	0.10	0018250840; Instrumentation Laboratory Werfen, Milano, Italy	
Cholesterol	1.38	1.56	0.10	0018250540; Instrumentation Laboratory Werfen, Milano, Italy	
Urea	1.23	1.14	0.01	0018255440; Instrumentation Laboratory Werfen, Milano, Italy	
Calcium	1.86	0.93	0.20	0018250340; Instrumentation Laboratory Werfen, Milano, Italy	
Zinc	3.83	1.76	0.500	439-14906; Wako Chemicals GmbH, Neuss, Germany	
Ceruloplasmin	3.57	1.39	0.100	-	(Sunderman and Nomoto, 1970)
Total proteins	2.87	1.07	10.00	0018251440; Instrumentation Laboratory Werfen, Milano, Italy	
Albumins	3.41	1.20	16.00	0018250040; Instrumentation Laboratory Werfen, Milano, Italy	
AST/GOT	3.55	1.78	1.00	0018257540; Instrumentation Laboratory Werfen, Milano, Italy	
GGT	4.42	2.27	2.00	0018257640; Instrumentation Laboratory Werfen, Milano, Italy	
Bilirubin	2.46	3.11	2.00	0018254640; Instrumentation Laboratory Werfen, Milano, Italy	
Haptoglobin	3.50	3.64	0.01	-	(Skinner et al., 1991)
NEFA	5.00	2.27	0.010	434-91795 NEFA-HR (2) R1 Set; Wako Chemicals GmbH, Neuss, Germany	
BHB	3.42	0.82	0.100	RB1007; Randox Laboratories Ltd., Crumlin, Co. Antrium, UK	
Creatinine	3.09	1.09	18.00	0018255540; Instrumentation Laboratory Werfen, Milano, Italy	
ROMt	2.93	1.40	3.20	MC003; Diacron srl, Grosseto, Italy	
Paraoxonase	4.60	1.38	1.20	-	(Ferré et al., 2002)

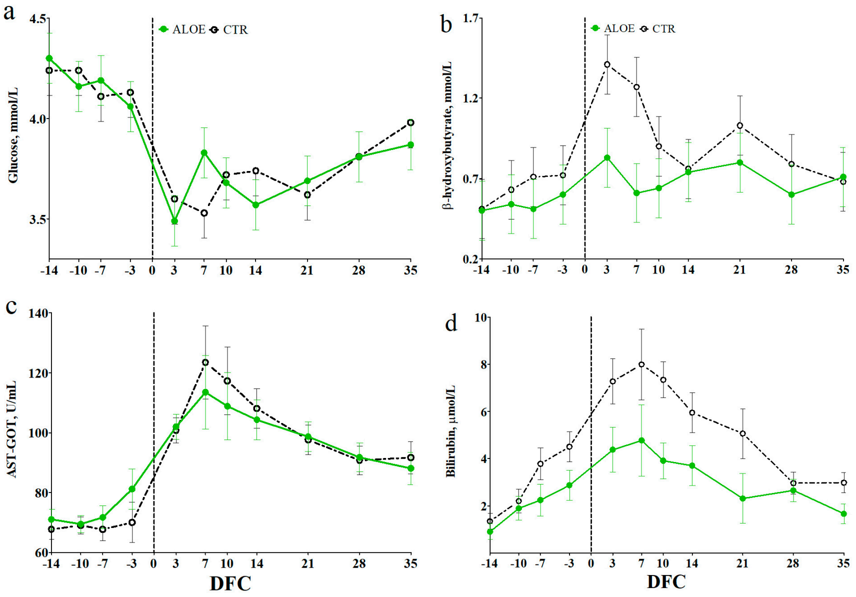

Supplementary File 2. Pattern of plasmatic concentrations of glucose (a), beta hydroxybutyrate (b), glutamate-oxaloacetate transaminase (AST-GOT; c) and bilirubin (d) expressed as their original values in dairy cows receiving placebo water (CTR; black dotted line) or cows receiving a drench of 200 g/d of *Aloe arborescens Mill.* whole plant homogenate between -14 and 14 days from calving (AAM; green solid line). DFC is days from calving.

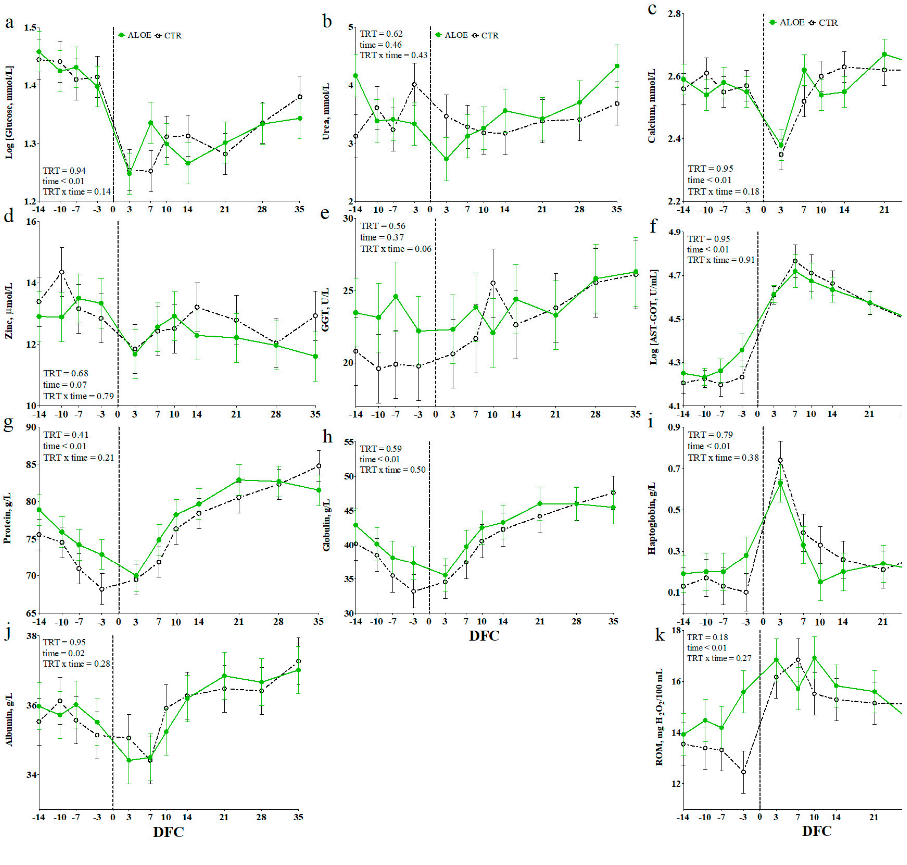

Supplementary File 3. Pattern of plasmatic concentrations of glucose (a), urea (b), calcium (c), zinc (d), gamma-glutamyl transferase (GGT; e), glutamate-oxaloacetate transaminase (AST-GOT; f), protein (g), globulin (h), haptoglobin (i), albumin (j) and reactive oxygen metabolites (ROM; k) in dairy cows receiving placebo water (CTR; black dotted line) or cows receiving a drench of 200 g/d of *Aloe arborescens Mill.* whole plant homogenate between -14 and 14 days from calving (AAM; green solid line). Log indicates data expressed as log-transformed; DFC is days from calving.
